# Molecular markers of anti-malarial drug resistance in southwest Ethiopia over time: regional surveillance from 2006 to 2013

**DOI:** 10.1186/s12936-015-0723-2

**Published:** 2015-05-19

**Authors:** Alexander Heuchert, Nuredin Abduselam, Ahmed Zeynudin, Teferi Eshetu, Thomas Löscher, Andreas Wieser, Michael Pritsch, Nicole Berens-Riha

**Affiliations:** Division of Infectious Diseases and Tropical Medicine, Medical Center of the University of Munich (LMU), Leopoldstrasse 5, 80802 Munich, Germany; Department of Laboratory Sciences and Pathology, Jimma University, Jimma for Infection Research (DZIF) at LMU, Munich, Germany; German Center for Infection Research (DZIF), Partner site Munich, Munich, Germany; Max von Pettenkofer-Institute of Hygiene and Medical Microbiology, Munich, Germany

## Abstract

**Background:**

Drug resistance is one of the main reasons of anti-malarial treatment failures and impedes malaria containment strategies. As single nucleotide polymorphisms (SNPs) have been found to correlate with anti-malarial drug resistance, the surveillance strategy includes continuous monitoring of known molecular markers and detection of new mutation patterns. With the introduction of artemisinin-based combination therapy, selection of specific patterns has been observed worldwide.

**Methods:**

From March to June 2013, whole blood was collected on filter paper from microscopically malaria positive patients in Jimma zone (District), southwestern Ethiopia. *Plasmodium falciparum*, *Plasmodium vivax* and mixed infections were included. SNPs were investigated by conventional or real-time PCR, restriction fragment length pattern analysis or sequencing. Results were compared to molecular patterns from Ethiopian isolates in 2004, 2006 and 2008/9.

**Results:**

*Plasmodium falciparum*, *P. vivax*, and mixed infections were molecularly confirmed in 177, 80, and 14 samples, respectively. In *P. falciparum*, mutations in the *pfcrt*, *pfmdr 1*and *pfATP 6 (SERCA)* gene were investigated. Whereas the mutation in the *pfcrt* gene at codon 76 K was still found in 95.6 % of all samples, the *pfmdr 1* 86 T mutation fell to 1.2 % (2/163) in 2013 compared to 9 % in 2008/9 and 86 % in 2006 (*P* <0.001). The *pfmdr 1* 184 F mutation dominated with 100.0 % (172/172) in 2013. Sequencing of the recently reported PF3D7_1343700 kelch propeller domain showed no mutation at codon 476. First sequencing data of the *pvmdr 1* gene from Jimma region revealed a prevalence of the mutations 976 F and 1076 L in 72.7 % (16/23) and 100.0 % (19/19) of the isolates, respectively.

**Conclusion:**

Since the introduction of artemether-lumefantrine (AL) in Jimma, Ethiopia, in 2006, the prevalence of certain SNPs associated with AL use has increased. Markers for chloroquine resistance in *P. vivax* were highly frequent. Continuous molecular and clinical surveillance are of paramount importance.

**Electronic supplementary material:**

The online version of this article (doi:10.1186/s12936-015-0723-2) contains supplementary material, which is available to authorized users.

## Background

The rise of drug-resistant parasites threatens to hamper malaria containment strategies. After the introduction of artemisinin-based combination therapy (ACT) in Jimma zone in 2006 [1], a certain shift in the distribution of wild types and mutations of defined single nucleotide polymorphisms (SNPs), associated with anti-malarial drug resistance, was expected.

The *Plasmodium falciparum* chloroquine resistance transporter (*pfcrt*) gene on chromosome 7 encodes a transmembrane protein to be found in the digestive vacuole of the parasite and has primarily been associated with resistances to chloroquine (CQ). However, it also seems to influence artemisinin, quinine and amodiaquine susceptibility [2–4]. The *P. falciparum* multi-drug resistance protein 1 (*pfmdr 1*) gene, located on chromosome 5, encodes a P-glycoprotein homologue 1 [5, 6]. Point mutations in codon 86 and 184 seem to be involved in artemether-lumefantrine (AL) resistance in some [7–10] but not all studies [11]. Concerning CQ, the PfMDR 1 protein is believed to add a modulatory effect to resistance mechanisms without conferring actual resistance [12]. An increased copy number has been linked to affect mainly mefloquine, but also artesunate, dihydro-artemisinin, halofantrine, quinine, and lumefantrine susceptibility [13–15]. Increased copy numbers of this gene however, do appear to play a minor role on the African continent [8, 9, 16].

The *P. falciparum* SERCA-type ATPase 6, encoded by the *pfATP 6* gene, was suggested to be the cytosolic target structure of artemisinins [17–19]. A variety of polymorphisms located in the *pfATP 6* gene have been reported [20–23]. However, their actual importance concerning artimisinin resistance remains unclear. Recently, mutations in the *K13-propeller* gene encoding the PF_1343700 kelch propeller domain have been proposed to be determinants for artemisinin resistance [24]. Yet its actual function within the parasite organism is unknown and can only be assumed, as homologous proteins in other organism can be found participating in a wide variety of pathways [24–26].

The *P. vivax* multi-drug resistance protein1, encoded by the *pvmdr 1* gene, is the *pfmdr 1* orthologue in *P. vivax* and is presumed to be connected to changes of CQ, amodiaquine and sulfadoxine-pyrimethamine treatment response. Specifically mutations in codon 976 and 1076 seem to be responsible for the aforementioned associations [27–29].

In this present study, the prevalence of these molecular markers in southwestern Ethiopia collected in 2013 was compared to earlier published and partially unpublished data from 2004, 2006 and 2009 from this region [30–32].

## Methods

### Study site and patient material

Sample collection took place from March until June 2013 in five different health centres in Goma province around Jimma town, approximately 355 km southwest of the Ethiopian capital Addis Ababa. Malaria transmission is seasonal with minor transmission from April to June and major transmission from September to December. The study was implemented in the routine diagnostic settings. When malaria was clinically suspected, microscopic investigations of blood slides were performed. Slides were stained with Giemsa and independently read by two experienced microscopists. Only microscopically malaria-positive patients were considered for inclusion in the study. *Plasmodium falciparum* and *P. vivax* single infections as well as mixed infections were included in the study. Pregnant women and children under the age of one year were excluded. Ethical clearance was granted by the Ethical Committee of the Universitiy of Jimma, Ethiopia, as well as of the University of Munich, Germany. A signed and dated informed consent form was obtained from each patient or parent/guardian as applicable. Blood samples for molecular investigations were derived from fingerprick samples applied on filter paper (Standard-Whatman Cellulose Chromatography paper 3MM, GE Healthcare, Fairfield, CT, USA) after recruitment. Filter papers were air-dried, individually packed in sterile DNA free zipper bags and stored in Jimma at ambient temperature. After arrival in Munich, samples were stored frozen at −20 °C.

### DNA extraction and amplification

DNA was extracted from filter paper using the Chelex method as described earlier [31, 33]. For parasite specification, a nested conventional polymerase chain reaction (PCR) was used [34]. In order to survey the *pfcrt* 76 K mutation, a nested conventional PCR followed by enzyme digestion with ApoI was performed as described previously [2, 35]. The specific restriction fragment length pattern (RFLP) analysis enables to discriminate between mutation and wild type. The validity of the assay was confirmed by sequencing 10 % of the samples and comparing the obtained results with the RFLP result.

To analyse *pfmdr 1* mutations at codons 86 and 184, the real-time PCR described by Purfield *et al.* was established [36]. Again, the validity of the method was confirmed by sequencing of 10 % of the samples. *Pfmdr 1* copy numbers were investigated as previously described in more detail [14]. The method was thereby adapted for a Biorad real-time PCR cycler.

Gel electrophoresis was performed with DNA GelRed™ stained 1.25-2 % agarose gels. For RFLP discrimination, high-resolution agarose (Roth®) was used.

Sequencing was performed using Big Dye Terminator 3.1 and DyeEx® 2.0 Spin Kit as described earlier [31]. Amplified fragments from *pfATP 6*, *pvmdr 1* and the newly described *K13-propeller* gene were sequenced using the same primer sequences as used for PCR amplification. For all primer sequences refer to Additional file 1. Genomic sequences were analysed by the sequence alignment editors Bioedit and DNASIS MAX (version 3.0). Reference sequences were obtained from the National Center for Biotechnology Information (NCBI). Sequence similarity was investigated via Basic Local Alignment Search Tool (BLAST) [37]. (Reference sequences: *pfmdr 1* 3D7 AL844501.1, *pfcrt* XM_001348968.1, *pfATP 6* AB121059.1, *K13 propeller* XM_001350122.1, *pvmdr 1* EU333975.1,).

## Results

### Sample collection and species differentiation

Altogether 338 finger prick samples of microscopically positive malaria patients were collected. Seventeen (5.0 %) samples originated from Choche Health Centre, 24 (7.1 %) from Limu Shay Health Centre, 154 (45.6 %) from Didisa Health Centre, three (0.9 %) from Jimma Health Centre, and 140 (41.4 %) from Shanan Gibe Hospital. *Plasmodium falciparum* and *P. vivax* could be detected by microscopy in 197 (61.2 %) and 98 (30.4 %) samples, respectively. A total of 27 (8.4 %) samples were classified as mixed infections with both species. For 16 samples, microscopic data were missing.

Molecular species differentiation by nested PCR confirmed 177 samples of *P. falciparum*, 80 samples of *P. vivax* and 14 mixed samples. A 67 of 338 (19.8 %) samples remained molecularly malaria-negative. Repetition and increase in DNA template resulted in the same result. Prevalence of *P. falciparum* and *P. vivax* mono-infections in these 271 positive samples were 65.3 % and 29.5 %, respectively.

### Molecular patterns in *Plasmodium falciparum*

In *P. falciparum* the following codons were assessed, mixed infections were included in the analysis: *pfcrt* 76, *pfmdr1* 86 and 184, *pfATP 6* codons 263–431 and the recently published K13-propeller domain with codon 476. The amplification and enzyme digestion of the *pfcrt* amplicon was successful for 159 samples. Within these, 152 (95.6 %) samples showed the mutation at codon 76 T, whereas only seven showed wild type (76 K) characteristics (Table 1). No mixed infections (wild type and mutation in one sample) could be detected. Sequencing 10 % of the samples revealed that both, the CVIET and CVMNT haplotype are present in 50 % of cases in codons 72–76. Interestingly, in 2009 the CVIET haplotype could be found in all cases.

**Table 1:** Prevalence of wild type and mutation in different genes in P. falciparum and P. vivax in Jimma, Ethiopia 2013

Gene	Codon	Wild type	Mutation
		N (%)	N (%)
*Plasmodium falciparum*			
*K13-propeller*	M476I	25/25 (100.0)	0/25 (0)
	N531I	24/25 (96.0)	1/25 (4.0)
*pfATP 6*	L402V	32/32 (100.0)	0/32 (0.0)
	E431K	39/48 (81.2)	9/48 (18.8)
*pfmdr 1*	N86Y	161/163 (98.8)	2/163 (1.2)
	Y184F	0/171 (0.0)	171/171 (100.0)
*pfcrt*	K76T	7/159 (4.4)	152/159 (95.6)
*pfcrt + pfmdr* ^*1*^	K76T, N86Y	5/159 (3.1)	1/159 (0.6)
*Plasmodium vivax*			
*pvmdr*	Y976F	6/22 (22.3)	16/22 (72.7)
	F1076L	0/19 (0)	19/19 (100.0)

^1^Showing both wild type or both mutation

Concerning the *pfmdr 1* gene, only two samples were identified as *pfmdr 1* 86Y mutants. The prevalence of the 86 N dominated with 98.8 % (n = 161). The *pfmdr 1* Y184F haplotype was found in all 171 amplified samples (Table 1). No mixed infections were observed. *Pfmdr* copy numbers were only investigated in 2009, all samples showed only one copy of the gene.

Sequencing of the *pfATP 6* gene was performed from 50 randomly chosen samples, the *K13- propeller* region was sequenced from 25 randomly selected samples. The *pfATP 6* 431 K mutation showed a prevalence of 18.8 % (9/48). One sample failed to amplify properly, another sample was not distinguishable by sequencing results (Table 1). Given that the sequences often were too short to allow full judgement, no other mutations could be confidently proven.

As the *K13-propeller* gene is uncommonly long for the sequencing method used, only sequences from codon 343 to codon 693 could reliably be assessed. No mutations including M476I as described in Ariey *et al.* [24] were detected. However, one mutation at codon 531 (N531I) was identified that had not been described earlier (Table 1).

There was no significant difference in the distribution of molecular patterns between the different health centres.

### Molecular patterns in *Plasmodium vivax*

Sequencing results of the *pvmdr1* gene have not been reported before from this region.

From 24 out of 25 randomly chosen samples, a partial *pvmdr1* sequence could be successfully amplified and sequenced. The homology with the reference strain (EU333975.1) was 100 % for the wild type. The sequence proved to be highly conserved on a global level. There was no difference in similarity between regionally closer isolates compared to isolates from other continents apart from the formerly described mutations at codon 976 and 1076. Prevalence of the Y976F mutation was 72.7 %, all readable samples showed the F1076L mutation. Two and five samples were indistinguishable for codon 976 and 1076, respectively. No other mutations were found in these sequences spanning from codon 938 to 1086. There was no significant difference in the distribution of molecular patterns between the different health centres. Two exemplary sequences are published at GenBank [38].

### Mutations over time

**Fig. 1:**
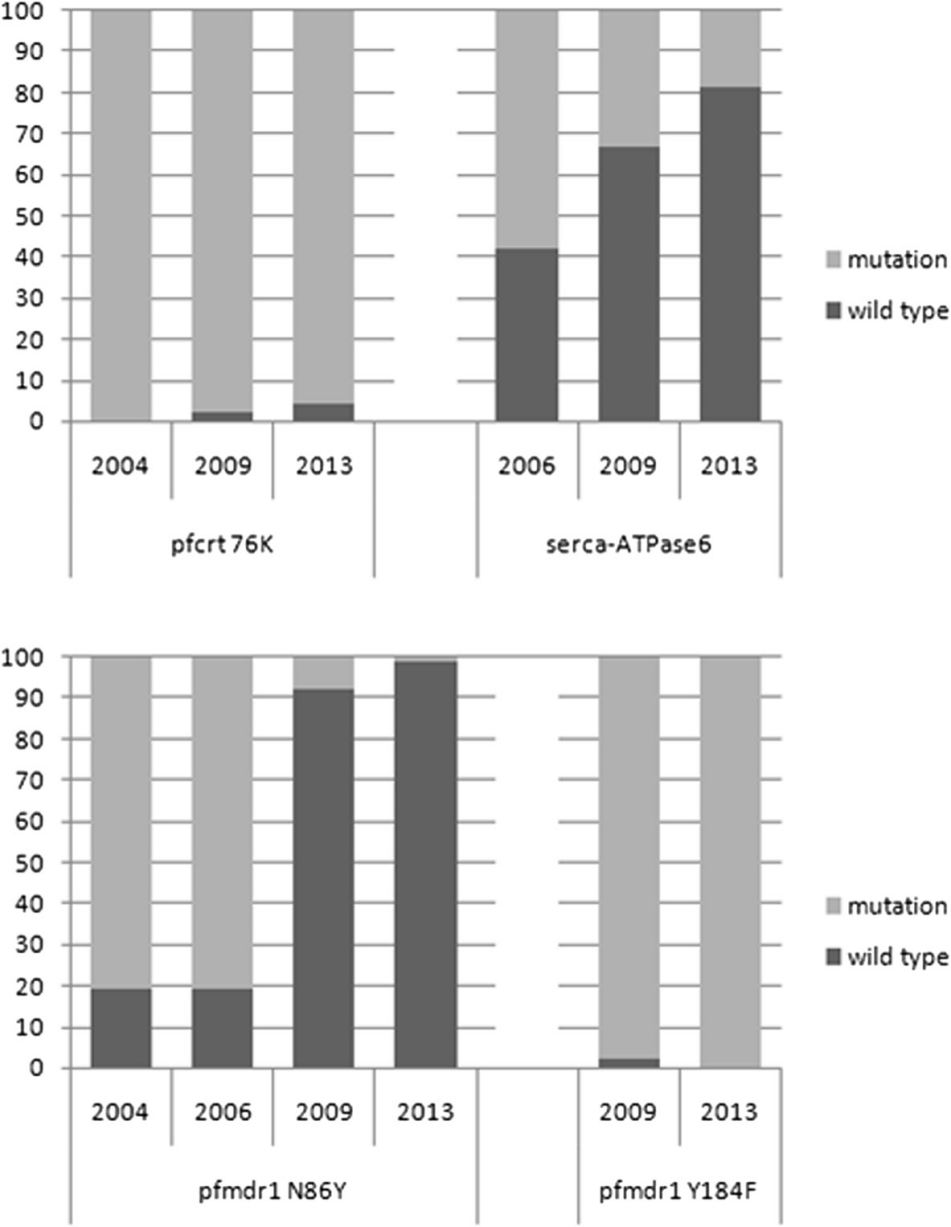
Prevalence of investigated mutations over time. Alterations of molecular markers with regard to the introduction of ACT in Jimma zone in 2006. No significant change in the *pfcrt* gene sequence was observed. The *SERCA* 431 K mutation dropped from 58.3 % in 2006 to 18.8 % in 2013 but the sample size was very low. Changes in the *pfmdr 1* gene were remarkable. In 2006 the mutation at codon 86Y was dominant with 80.6 %. Mixed infections were taken together with wild type samples. The wild type alone was only detectable in 14.3 %. In 2009 and 2013, only 9.2 % and 1.2 % showed the mutation 86Y, respectively. The prevalence of *pfmdr 1* 84 F was high in 2009 and remained dominant. Data from 2004 are taken from reference 30

Mutations in the following *P. falciparum* genes were investigated over time: *pfcrt*, *pfmdr,* and *pfATP 6* (Fig. 1). The sample size was 98 and 251 in 2006 and 2009, respectively. Mutation analysis of the *pfcrt* and *pfmdr 1* gene was performed for all samples. Confirmative sequencing was conducted for 10 % of these samples in 2009. On average, 15 % and 10 % of all samples were successfully sequenced at the *pfATP 6* gene in 2006 and 2009, respectively.

There was no significant change in the *pfcrt* gene observed. Prevalence of the wild type remained negligible (4.4 %). However, changes in the *pfmdr 1* gene were remarkable. Until 2006, the mutation at codon 86Y was dominant: 80.6 % (79/98) of the samples showed the mutation, only 14.3 % presented the wild type, the rest were mixed clone infections presenting both haplotypes [31]. In 2009, the distribution had changed dramatically. Only 9.2 % showed the mutation 86Y and 228 of the 251 samples (90.8 %) presented the wild type 86 N [32]. In 2013, the prevalence of 86Y dropped to as low as 1.2 %. *Pfmdr 1* 184 F was first investigated in 2009, the prevalence was 98 %, increasing to 100 % in 2013. The *pfATP 6* 431 K mutation dropped from 58.3 % (7/12) in 2006 to 18.8 % (9/48) in 2013 but the sample size was very low. Other mutations described in this short sequence were not detectable.

## Discussion

In this study, molecular resistance patterns in Ethiopian *P. falciparum* and *P. vivax* isolates were investigated. *Pfcrt* 76 T, associated mainly with CQ resistance, could be found almost as frequently as before introduction of ACT. Either continuous CQ use remains common in Jimma region, or the *pfcrt* wild type provides no fitness advantage under AL treatment. As the wild type 76 K re-emerged under ACT and in absence of CQ in some countries [39], continuous CQ treatment in Jimma region should be at least suspected. CQ is not recommended nor released in the health centres for *P. falciparum* in Jimma region but available on the free market and recommended for *P. vivax* treatment*.*

As shown above, microscopic diagnosis was not always reliable; performance was similar in all health centres. Only in 203 (60.1 %) of all samples, the microscopy result matched the PCR result. A total of 16 samples microscopically diagnosed as vivax malaria could be proven to be *P. falciparum* infections by PCR. Overall, 19.8 % microscopically positive samples were negative by PCR, 21 were microscopically categorized as vivax malaria and treated accordingly. Therefore, *P. falciparum* parasites still have opportunities to get in contact with CQ. Also, self-treatment of the rural population in less severe cases seems to be still very common as CQ is cheap and easily available. The prevalence of *P. vivax* in Jimma region is between 30-70 % [40]. The performance data are consistent with a recent study from Ethiopia assessing malaria diagnostic capacities in Ethiopian health centres [41]. A recent study from Mozambique showed a very similar changing pattern. The *pfmdr 1* allele N86 increased from 19.5 % in 2003–2005 to 73.2 % in 2010–2012 after introduction of AL [42]. Interestingly, a recent study from southeast Ethiopia (Omo Nada, Bala Wajo, Arba Minch and Harar) showed different results [43]. The authors declared a consequent absence of CQ use in this area. Prevalence of 76 T was 13.5 % (23/170) in the South and 32 % (8/25) in the East. Overall, the C72S mutation was observed only in 3.6 %.

The authors further stated that the CQ-sensitive CVMNK haplotype was found in 95.9 %, the mutant haplotype SVMNT in 4.1 % and CVIET was absent at codons 72–76. Sequencing of the mutant samples from Jimma showed in 100 % the CVIET haplotype in 2009 and 50 % CVIET / 50 % CVMNT in 2013, in accordance with other studies from Africa [32]. The C72S mutation was absent as well as the sensitive CVMNK haplotype. However, a shift to CVMNT might be a possible interpretation. These regional differences are highly interesting and warrent further investigation.

The *Pfmdr 1* wild type 86 N was frequently detected in areas where AL is used. A selection for or re-introduction of the wild type is discussed for artemether as well as lumefantrine and might be the explanation for this drastic change [7–10]. Since the introduction of AL in Jimma zone in 2006, the prevalence of the wild type remarkably increased (*P* <0.001, data from 2006 compared to 2013 by Wilcoxon rank-sum test; Fig. 1). Comparing the *pfmdr1* 184 haplotype with data from 2009, the prevalence of the mutation remained stable at above 98 %. These findings support the theory that the *pfmdr1* 184 F is selected under drug pressure, especially under AL treatment [9]. Unfortunately, no earlier data exists for *pfmdr 1* Y184F from this area. A recent study from southeast Ethiopia reported a prevalence of 9 % for N86 [40]. This is similar to the data observed in this study. The prevalence of Y184F was 5 % only compared to 100 % in this study. Different selection mechanisms might be responsible. The diagnostic tool used in this study consisted of a real-time PCR with selective probes, sequencing was only performed in 10 % of the samples but the latter consistently confirmed the results of the PCR.

In 2009, all samples from Jimma showed only one *pfmdr* copy. In the above-mentioned study from southeast Ethiopia, all samples presented also with only one copy [43]. This is in accordance with other studies from Africa [8, 9, 16].

The prevalence of the E431K mutation in the *pfATP 6* gene dropped from 58.3 % in 2006 [31] to 18.8 % in 2013. Recently, E431K together with A623E has been described to reduce artimisinin susceptibility [20]. The A623E mutation was extremely rare with 1/24 (4.2 %) in 2006 and 0/33 (0 %) in 2009. No samples with both mutations could be detected. The mutation was not assessed in 2013 but the decline of the E431K mutation contradicts selection of it under artemisinin pressure.

The new potential candidate referring resistance to artemisinins, the *K13 propeller* gene, showed no mutations at codon 476. This mutation has been described by Ariey *et al.* in highly resistant samples from Cambodia [24]. A new mutation at codon N531I was found in only one isolate. Clinical effectiveness of AL was still 95 % in 2009. Clearance rates showed no significant prolongation, no resistance could be suspected then. More recent clinical data from this area were not available [40].

Sequencing of the *pvmdr 1* gene revealed a high prevalence of the Y976F and F1076L mutations. This correlates well with the long lasting CQ use in this area. Treatment failures were reported in recent *P. vivax* studies [43–46]. Comparison of the Ethiopian *pvmdr 1* sequences to sequences from Brazil (AY571984.1), Cambodia (JQ925836.1), India (KC818412.1), Korea (GU476519.1, GU244390.1) Madagascar (EU683815.1) and Thailand (KC121338.1) showed highly conserved features without systematic geographical clustering.

## Conclusion

The prevalence of SNPs associated with drug resistance has been influenced since the introduction of AL treatment in Ethiopia in 2006 (*P* <0.001). The wild type of *pfmdr* codon 86 seems to possess a selection advantage. No significant change was observed for the *pfcrt* codon 76. First sequencing data of the *K13 propeller* region revealed no mutations as described in Asia. Mutations associated with CQ resistance in *pvmdr 1* were highly prevalent. Continuous molecular and clinical surveillance are of paramount importance.

## Additional file

Additional file 1:
**Primers used in the study.**
