# Supplementary material for: Molecular markers of anti-malarial drug resistance in southwest Ethiopia over time: regional surveillance from 2006 to 2013
Source: Malar J. 2015 May 19;14:208. doi: 10.1186/s12936-015-0723-2 (PMC4490604; doi:10.1186/s12936-015-0723-2)
Supplement: Additional file 1: — Primers used in the study. [file 12936_2015_723_MOESM1_ESM.docx]

**Additional Table 1. Primers used in the study**

| **Target** | **Primer** | **Publication** |
| --- | --- | --- |
| Species | Nest 1 *Plasmodium* genus  Forward: rPLU5: 5’-CCTGTTGTTGCCTTAAACTTC-3’  Reverse: rPLU6: 5’-TTAAAATTGTTGCAGTTAAAACG-3’  Nest 2 *Plasmodium falciparum*  rFAL1: 5’-TTAAACTGGTTTGGGAAAACCAAATATATT-3’  rFAL2: 5’-ACACAATGAACTCAATCATGACTACCCGTC-3’  Nest 2 *Plasmodium vivax*  rVIV1: 5’-CGCTTCTAGCTTAATCCACATAACTGATAC-3’  rVIV2: 5’-ACTTCCAAGCCGAAGCAAAGAAAGTCCTTA-3’  Nest 2 *Plasmodium ovale*  rOVA1: 5’-ATCTCTTTTGCTATTTTTTAGTATTGGAGA-3’  rOVA2: 5’GGAAAAGGACACATTAATTGTATCCTAGTG-3’  Nest 2 *Plasmodium malariae*  rMAL1: 5’-ATAACATAGTTGTACGTTAAGAATAACCGC-3’  rMAL2: 5’-AAAATTCCCATGCATAAAAAATTATACAAA-3’ | Snounou et *al*. 1999 [34] |
| PfATP 6 | Forward: ATP1: 5'- CCGCTATTGTATGTGGTAGATG-3'  Reverse: ATP2: 5'-ATTCCTCTTAGCACCACTCCT-3') | Menengon et *al*. 2008 [21] |
| K13-propeller domain | Nest1:  Forward: K13-1: 5’-CGGAGTGACCAAATCTGGGA-3’  Reverse: K13-4: 5’-GGGAATCTGGTGGTAACAGC-3’  Nest2:  Forward: K13-2: 5’-GCCAAGCTGCCATTCATTTG -3’  Reverse: K13-3: 5’-GCCTTGTTGAAAGAAGCAGA -3’ | Ariey et *al*. 2014 [24] |
| Pfcrt | Nest 1:  Forward: TCRP1: 5’-CCGTTAATAATAAATACACGCAG-3’  Reverse: TCRP2: 5’-CGGATGTTACAAAACTATAGTTACC-3’  Nest 2:  Forward: TCRD1: 5’-TGTGCTCATGTGTTTAAACTT-3’  Reverse: TCRD2: 5’-CAAAACTATAGTTACCAATTTTG-3’ | Djimde et *al*. 2001 [2] |
| Pfmdr 1 | Codon 86  Forward: 86F: 5’-TGTATGTGCTGTATTATCAGGAGGAAC-3’  Reverse: 86R: 5’AATTGTACTAAACCTATAGATACTAATGA TAATATTATAGG-3’  Asn (wt*) probe:  5’-6 FAM-ACCTAAAT**T**CATGTTCTTT-MGB-NFQ-3’  Tyr (mut**) probe:  5’-VIC-ACCTAAAT**A**CATGTTCTTT-MGB-NFQ-3’  Codon 184  Forward: 184F:  5’-AAGATGGACAATTTCATGATAATAATCCT-3’  Reverse: 184R:  5’-AATACATAAAGTCAAACGTGCATTTTTTA-3’  Tyr (wt) probe:  5’-6-FAM-CTTTTTAGGTTTAT**A**TATTTGGT-MGB-NFQ-3’  Phe (mut) probe:  5’-VIC-CTTTTTAGGTTTAT**T**TATTTGGT-MGB-NFQ-3’ | Purfield et *al*. 2004 [36] with modifications |
| Pfmdr 1 copy numbers | pfmdr1–1F 5’-TGC ATC TAT AAA ACG ATC AGA CAA A-3’  pfmdr1–1R 5’-TCG TGT GTT CCA TGT GAC TGT-3’  pfmdr1-probe:  5’-TTT AAT AAC CCT GAT CGA AAT GGA ACC TTT G-3’  β-tubulin-1F 5’-TGA TGT GCG CAA GTG ATC C-3’  β –tubulin-1R 5’-TCC TTT GTG GAC ATT CTT CCT C-3’  β –*tubulin*-probe:  5’-TAG CAC ATG CCG TTA AAT ATC TTC CAT GTC T-3’ | Price et *al*. 2004 [37] with modifications |
| Pvmdr 1 | Forward: PvmdrF: 5'-GCTGTCAGCACATATTAACAGAGA-3'  Reverse: PvmdrR: 5'-TGCCTACTGGTTTGGTTCC-3' | Inhouse design |
